# CD39/CD73/A2AR pathway and cancer immunotherapy

**DOI:** 10.1186/s12943-023-01733-x

**Published:** 2023-03-02

**Authors:** Chenglai Xia, Shuanghong Yin, Kenneth K. W. To, Liwu Fu

**Affiliations:** 1 Affiliated Foshan Maternity and Child Healthcare Hospital, Southern Medical University, Foshan, 528000 China; 2 School of Pharmaceutical Sciences, Southern Medical University, Guangzhou, 515150 China; 3 State Key Laboratory of Oncology in South China, Collaborative Innovation Center for Cancer Medicine, Guangdong Esophageal Cancer Institute, Sun Yat-sen University Cancer Center, Guangzhou, 510060 China; 4 School of Pharmacy, The Chinese University of Hong Kong, Hong Kong, China

**Keywords:** Immunosuppressive tumor microenvironment, Cancer immunotherapy, CD39, CD73, Adenosine receptor, A2AR

## Abstract

Cancer development is closely associated with immunosuppressive tumor microenvironment (TME) that attenuates antitumor immune responses and promotes tumor cell immunologic escape. The sequential conversion of extracellular ATP into adenosine by two important cell-surface ectonucleosidases CD39 and CD73 play critical roles in reshaping an immunosuppressive TME. The accumulated extracellular adenosine mediates its regulatory functions by binding to one of four adenosine receptors (A1R, A2AR, A2BR and A3R). The A2AR elicits its profound immunosuppressive function via regulating cAMP signaling. The increasing evidence suggests that CD39, CD73 and A2AR could be used as novel therapeutic targets for manipulating the antitumor immunity. In recent years, monoclonal antibodies or small molecule inhibitors targeting the CD39/CD73/A2AR pathway have been investigated in clinical trials as single agents or in combination with anti-PD-1/PD-L1 therapies. In this review, we provide an updated summary about the pathophysiological function of the adenosinergic pathway in cancer development, metastasis and drug resistance. The targeting of one or more components of the adenosinergic pathway for cancer therapy and circumvention of immunotherapy resistance are also discussed. Emerging biomarkers that may be used to guide the selection of CD39/CD73/A2AR-targeting treatment strategies for individual cancer patients is also deliberated.

## Introduction

Immune homeostasis refers to the tightly regulated balance of immune activation and suppression in our body. While it ensures efficient pathogen recognition and destruction during infection, it prevents excessive and inappropriate self-targeting immune reactions. The accumulating evidences indicate that the majority of cancers are closely associated with failure of this immune homeostasis [1]. Under normal physiological conditions, immune checkpoints play crucial role to protect tissues from damage when the immune system is producing an inflammatory response to fight against pathogenic infection. In cancer cells, the immune checkpoint pathways are highly active and they allow the tumors to evade the antitumor immune response [2]. Immune checkpoint molecules, including inhibitory and stimulatory immune checkpoint molecules, are defined as ligand-receptor pairs that exert inhibitory or stimulatory effects on immune responses, which expresses on immune cells, antigen-presenting cells, tumor cells, or other types of cells, mediating the progress of the adaptive immune system, in particular, T cells and innate immune system. The number of immune checkpoints is increasingly discovered, like PD-1(programmed cell death protein 1), PD-L1(programmed cell death-Ligand 1), LAG3(LymphocyteActivation Gene-3), B7-H3(CD276, Recombinant Cluster Of Differentiation 276), TIM3(T cell immunoglobulin domain and mucin domain-3) [3]. To escape from neoantigen induced antitumor immunity, pathways regulating immune checkpoints are hijacked by tumor cells to induce TIL (Tumor Infiltrating Lymphocyte) exhaustion or suppression. Such as PD-1 and CTLA-4, expressed on activated T cells lead to inhibition of T-cell activation upon binding to their ligands on tumor cells/antigen-presenting cells [4]. The development of immune checkpoint blockade therapy represents a major breakthrough in cancer therapy by unleashing the latent antitumor immune response [5].

In recent years, novel strategies targeting the tumor microenvironment (TME) have emerged as promising therapeutic approaches for cancer treatment [6]. However, while immune checkpoint blockade therapy could produce substantial anticancer effect and durable remission in a small proportion of cancer patients, most patients did not respond due to the presence of immunosuppressive TME [7]. Extracellular adenosine (eADO) activates cell signaling pathways through one of the four known G-protein-coupled adenosine receptors A1, A2A, A2B, and A3. A2A receptors are G-protein-coupled stimulatory pathways that are up-regulated in response to immune cell activation [8]. A2A receptor is a high-affinity receptor expressed on T cells and natural killer T (NKT) cells, monocytes, macrophages, DC (Dendritic cells) and natural killer (NK) cells. A2AR is up-regulated in macrophages in response to NF-κB, STAT1 and PPARγ as well as adenosine signaling, and A2AR activation inhibits the secretion of neutrophil chemokines, thereby reducing the inflammatory response. In effector T cells, increased PKA activity secondary to A2aR signaling has a lots of inhibitory effects, including 1) Inhibiting multiple MAP kinases (ERK1 and JNK); 2) Inhibition of protein kinase C activity, which is important for effector cell activation; 3) Activation of CREB-mediated inhibition of NF-κB and activated T nuclear factor (NF-AT) [9]. Finally, A2AR signal transduction on effectors and regulatory T cells triggers increased expression of other immune checkpoint pathways, including PD-1, CTLA-4(cytotoxic T lymphocyte-associated antigen-4), and LAG-3(lymphocyte activation gene 3) [9]. Thus, the A2AR signal may represent a novel checkpoint pathway. What’s more, the production of adenosine in inflamed tissues combines the regression of inflammation in response to tissue damage with the deep suppression of the immune response by signaling the A2A receptor. However, this combination of wound-healing and immunosuppression is maladaptive in malignancies and is the basic mechanism of cancer immune evasion [10]. To this end, adenosine signaling represents a key metabolic pathway that impairs immunological surveillance [11].

**Fig. 1:**
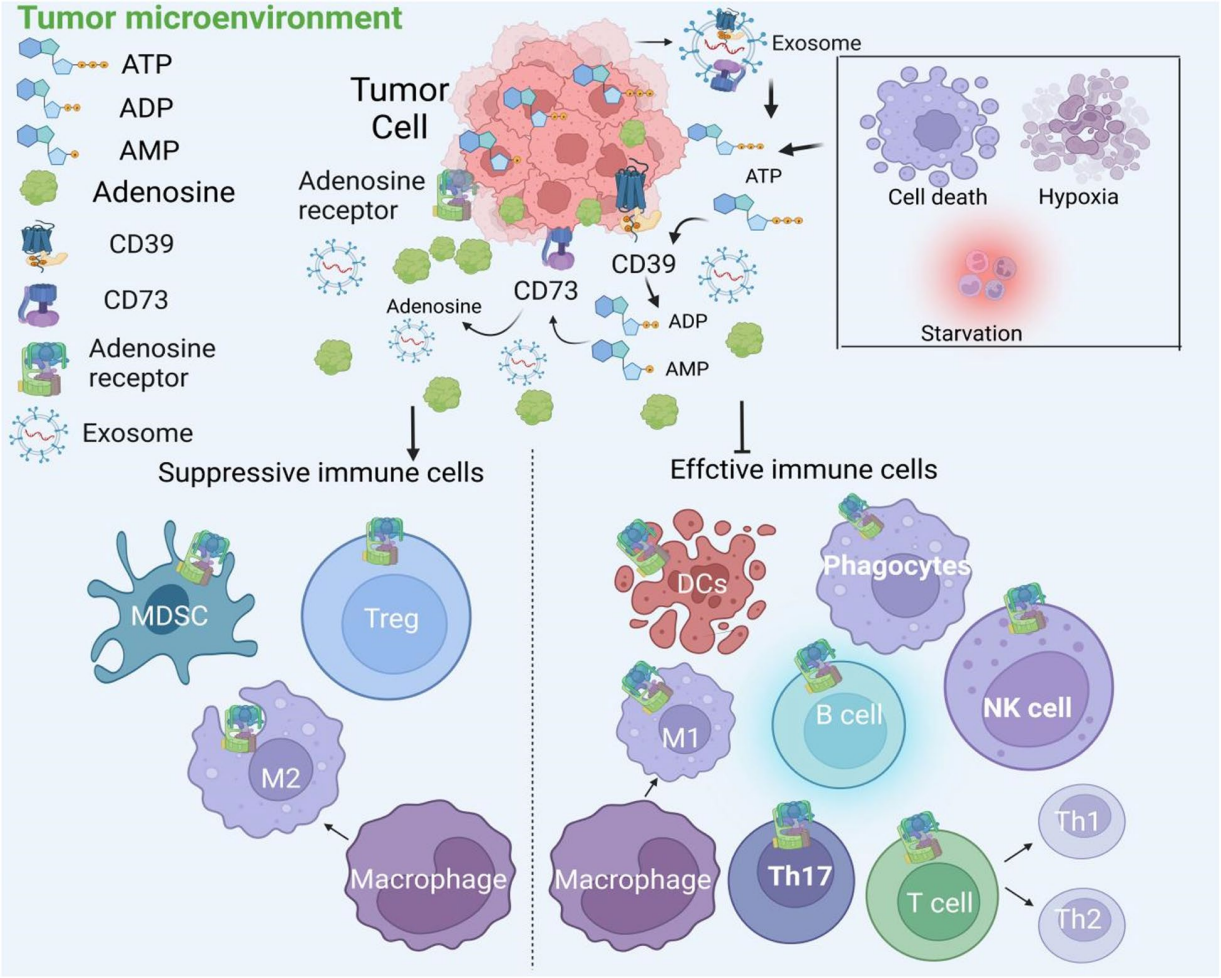
The two ectonucleotidases CD39 and CD73 control the metabolic fate of ATP and adenosine in the extracellular environment. Extracellular ATP is converted into its metabolites ADP and AMP sequentially by CD39, which is then further metabolized to adenosine by CD73. Activated CD39/CD73/A2AR signaling within the TME will suppress the function of antitumor immune cells (T cells, B cells, NK cells, and DCs) but promote the activity of the regulatory immune cells (MDSCs and Tregs), thus giving rise to a immunosuppressive TME. Notes: TME: tumor microenvironment; NK: natural killer; DCs: dendritic cells; MDSC: myeloid-derived suppressor cells; Treg: regulatory T cells; Th17: T helper 17 cells

Adenosine is an immunosuppressive metabolite produced at high concentration in TME that contributes to tumor-mediated immune evasion. Under normal conditions, adenosine and ATP are present at low levels in extracellular fluids [12]. The anticancer therapies are known to trigger the release of high levels of ATP to the extracellular compartments, which serves as a Danger-Associated Molecular Pattern (DAMP) to induce both innate and adaptive immune responses [13]. Extracellular ATP is dephosphorylated by ectonucleotidases (CD39 and CD73) to produce adenosine [14]. In contrast to extracellular ATP, adenosine is known to inhibit the activity of the effector immune cells but activate other immunosuppressive regulatory cells [15] (Fig. 1). Therefore, the extent of ATP release to the extracellular compartment and its degradation to adenosine should be limited to restrict the suppressive TME and to facilitate a durable antitumor immunity during cancer immunotherapy [16].

## CD39/CD73/A2AR Signalling within the TME

**Fig. 2:**
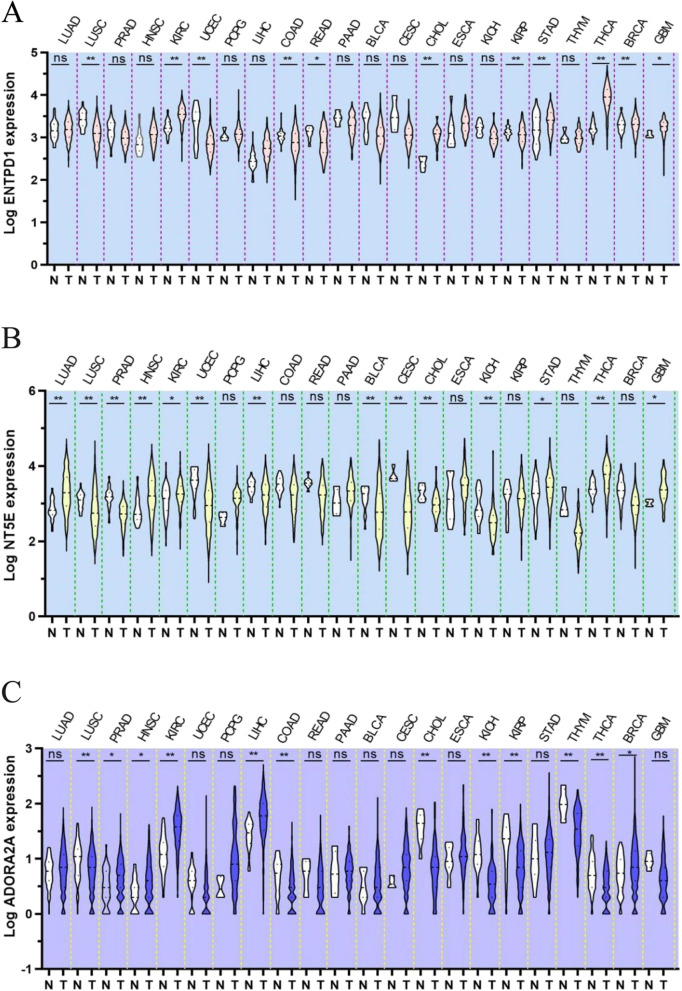
Gene-expression landscape of the three major components (CD39, CD73 and A2AR) in the adenosine signaling pathway in various solid cancer types. The Cancer Genome Altas (TCGA) analysis RNA-sequencing (RNA-seq) data of ENTPD1(**A**), NT5E (**B**) and ADORA2A (**C**), encoding the proteins CD39, CD73, A2AR, respectively, in human cancers. Notes: LUAD: lung adenocarcinoma; LUSC: Lung squamous cell carcinoma; PRAD: Prostate; HNSC: Head and Neck squamous cell; KIRC: Kidney renal clear cell carcinoma; UCEC: Uterinecorps Endometrial carcinoma; PCPG: Pheochromocytoma; LIHC: Liver hepatocellular carcinoma; COAD: Colon adenocarcinoma; READ: Rectum adenocarcinoma; PAAD: Pancreatic adenocarcinoma; BLCA: Bladder Urothelial Carcinoma; CESC: Cervical squamous cell carcinoma; CHOL: Cholangiocarcinoma; ESCA: Esophageal carcinoma; KICH: Kidney renal clear cell carcinoma; KIRP: Kidney renal papillary cell carcinoma; STAD: Stomach adenocarcinoma; THYM: Thyroid carcinoma; THCA: Thyroid carcinoma; BRCA: Breast invasive carcinoma; GBM: Glioblastoma multiforme. N = normal tissue; T = tumor specimen

CD39 and CD73 are highly expressed in various cell types within the TME (including tumor cells, stromal cells, endothelial cells, and the infiltrating immune cells) (Fig. 2) [17]. They are also known to be upregulated in response to the hypoxic tumoral environment. Moreover, both CD39 and CD73 are induced by Tregs (regulatory T cells) in response to adenosine signalling [18, 19], thereby setting up a feedback loop to maintain adenosine production and immunosuppression within the TME. A1R, A2AR and A3R have high affinity for adenosine whereas A2BR has low affinity for adenosine. Upon binding of adenosine to the A2AR or A2BR, cellular adenylyl cyclase activity is increased to raise intracellular cAMP (Cyclic Adenosine monophosphate) level, subsequently inhibiting antitumor immune responses and also activating immune suppressor cells [20, 21].

The CD39 protein (exonucleoside triphosphate diphosphate hydrolase 1; also known as NTPDase 1) has 510 amino acids, which harbors eleven cysteine residues and seven potential N-linked glycosylation sites [22]. There are two transmembrane domains in the CD39 protein. The cytoplasmic domain is relatively short whereas the extracellular domain is large and consists of five highly conserved segments that mediate the nucleotidase activity of the enzyme [23]. CD39 is localized on cell surface and it catalyzes the hydrolysis of extracellular nucleoside tri- and diphosphates to produce the corresponding monophosphates. It is noteworthy that glycosylation of CD39 plays a crucial role to ensure proper protein folding, cell membrane targeting and effective enzymatic activity [24]. The expression of CD39 is induced by a number of inflammatory cytokines, nutrient starvation, oxidative stress, and hypoxia stress via the action of a few transcription factors, including Sp1, Stat3, and the zinc finger protein growth factor independence-1 (GFI1) [25].

The CD73 protein (also known as ecto-5′-nucleotidase) is a glycosyl-phosphatidylinositol-linked cell membrane-bound enzyme found in most tissues [26]. It hydrolyzes the CD39-generated nucleoside monophosphates to the corresponding nucleosides [27]. In particular, CD73 is strongly linked with the generation of adenosine within the TME that stimulates cancer progression by suppressing antitumor immunity and promoting angiogenesis [28].

Extracellular adenosine could be produced by passive diffusion or active transport of intracellular adenosine [29]. On the other hand, it can also be generated by the enzymatic hydrolysis of extracellular ATP. In solid tumors, ATP is released into the extracellular space due to cell necrosis and other secretary mechanisms under the condition of hypoxia, inflammation, nutrient deprivation and cytotoxic drug treatment [30–32]. ATP released into the extracellular space is converted to AMP by CD39, and then AMP is further hydrolyzed to adenosine by CD73 [33]. Importantly, both CD39 and CD73 are highly expressed in the cell types within the TME (including tumor cells, immune cells, endothelial cells, and fibroblasts). Moreover, exosomes carrying CD39 and CD73 are constantly released from tumors to enrich the abundance of these ectonucleotidases within the TME. Recently, it has been found that cancer-derived exosomes carries CD39 and CD73 on the surface, and the exosomes from different types of cancer exhibit strong hydrolytic activity of ATP and 5 ‘amp- phosphate, which may be the mechanism that causes adenosine levels to rise in the tumor microenvironment [34–36]. Importantly, adenosine is known to suppress the activity of numerous immune cells including phagocytes, dendritic cells (DCs), NK cells (natural killer cells), T cells, B cells, Th17(T helper cell 17), macrophages, upon binding to the A2AR on their cell surface [17, 37]. On the other hand, adenosine can also promote the activity of a few regulatory and suppressive immune cells such as MDSCs (Myeloid-derived suppressor cells) and Tregs to dampen the antitumor immunity [38]. In addition, A2AR has been shown to inhibit macrophage activation by its downstream signaling. Adenosine-A2AR pathway could inhibit T-lymphocyte proliferation, activation, and cytokine production, leading to polarization of immunosuppressive T-regulatory cells [39]. As a result, blockade of A2AR offers a potential next-generation immune checkpoint mechanism for cancer immunotherapy [31].

Recent research has shown that adenosine suppresses immune responses in both CD4+ and CD8+ T cells by regulating the downstream signalling of A2AR [40–43]. Activation of A2AR by adenosine is known to suppress the proliferation and differentiation of naïve T cells, thus inhibiting Th1 and Th2 differentiation [44]. Moreover, high level of adenosine in the TME also disrupts CD8+ T-cell activation, expansion, and cytokine secretion to inhibit cytotoxic T-cell activity and interferes with NK cells cytolysis activity [37, 45].

B cells are the core component of the adaptive humoral immune system and they work by producing antigen-specific antibodies [46]. However, a growing body of research suggests that B cells could also regulate immune responses through mechanisms beyond antibody production [47]. Human B lymphocytes have been reported to express CD39, CD73, A1R, A2R, and A3R and they can also produce adenosine. The CD39(+)/CD73(+) B cells are capable of producing adenosine, which play critical role in regulating the immune responses of CD4+ and CD8+ T cells [48–50]. Human regulatory B cells (Bregs) express high levels of CD39 and they also release IL-10 to suppress T cell–mediated immune responses [51].

In human body, cancer immune surveillance is largely mediated by natural killer (NK) cells. They are effector lymphocytes of the innate immune system that target and kill tumor cells. NK cells are known to be regulated by various metabolic signaling including the purinergic pathway [52]. NK-cell maturation and antitumor immunity are regulated by adenosine signaling through A2AR. Extracellular adenosine interacts with adenosine receptors (predominantly A2AR) expressed on NK cells to mediate suppressive signals [53]. It has been demonstrated that conditional deletion of A2AR could increase the proportion of terminally mature NK cells at homeostasis and also in the TME [54]. Importantly, the specific targeting of A2AR on NK cells has been shown to delay tumor initiation and inhibit tumor growth in animal studies [55]. It is noteworthy that the combination of A2AR antagonists and NK cell–based therapies was shown to promote NK cell-mediated antitumor immunity [56–58].

Dendritic cells (DCs) represent the major antigen-presenting cells capable of initiating innate and adaptive immune responses to external pathogens and producing antitumor immunity. Apart from presenting antigens, they can also secrete various cytokines to regulate the immune responses [59, 60]. In DCs, CD39 can affect immunological synapses and intracellular signaling. High concentration of ATP was shown to increase indoleamine-2,3-dioxygenase and thrombospondin 1 levels, which subsequently leads to immunosuppression. The immunosuppressive effect of extracellular ATP and adenosine was related to the decreased secretion of proinflammatory cytokines by DCs [45].

It is commonly believed that regulatory T cells (Tregs) are the prime mediators of immune suppression and they are critical for maintaining peripheral tolerance. They play a key role in protecting against autoimmune diseases and reducing chronic inflammatory conditions, including asthma and inflammatory bowel disease. Besides this important physiological function, Tregs are also known to limit antitumor immunity [61]. To this end, Treg activity can be regulated by the CD39/CD73/A2AR pathway. The activation of adenosine receptor A2AR by extracellular adenosine on Treg cell surface has been shown to stimulate Treg cell proliferation to promote immunosuppression [62].

T helper 17 cells (Th17) are a subset of proinflammatory T helper cells, characterized by their production of interleukin 17 (IL-17). It has been shown that in vitro generated Th17 cells with the cytokines IL-6 and TGF expressed CD39 and CD73, thereby leading to adenosine release and suppression of CD4+ and CD8+ T effector cell functions [63]. On the other hand, the expression level of CD39 and CD73 is decreased in the proinflammatory M1 macrophages, but is increased in the anti-inflammatory M2 macrophages. Therefore, adenosine can indeed promote anti-inflammatory cytokine production but suppress pro-inflammatory cytokine production [64.]. Myeloid-derived suppressor cells (MDSCs) are a heterogeneous group of immature myeloid cells, which suppress T cell response. They are composed of the progenitors of DCs, macrophages, and granulocytes. In the TME, it has been shown that TGF-β and HIF-1α can regulate CD39 and CD73 expression in MDSCs [65].

The mechanism by which the CD39/CD73/adenosine-A2AR suppresses antitumor immunity within TME is depicted in Fig. 1. The immune system plays a vital role in suppressing the development and progression of tumor. Recent research reveals that high levels of immunosuppressive adenosine within the TME contributes substantially to cancer immune evasion. Therefore, the production of high concentration of extracellular adenosine within the TME is mediated by the CD39/CD73/adenosine pathway. The development of novel strategies for immunotherapy by inhibition of this adenosine/A2AR pathway will be discussed in the following sections.

## The expression and function of CD39/CD73/A2AR in various Cancer types

Tumor progression and metastasis are regulated by the cross-talk between tumor cells and the TME [66]. CD39 is expressed in infiltrating immune cells as well as on the cancer cells in a range of human cancers, including lung cancer, squamous cell carcinoma of the head and neck, clear cell carcinoma of the kidney, rectal adenocarcinoma, thyroid cancer, breast cancer, and multiforme glioblastoma solid tumors, studies have shown that high expression of CD39 is strongly associated with adverse outcomes [67]. Like CD39, the expression of CD73 in the tumor microenvironment has been studied as a prognostic biomarker for clinical outcomes of a variety of tumor types, including squamous cell carcinoma of the lung, pheochromocytoma, pancreatic cancer, urothelial carcinoma of the bladder, esophageal carcinoma, gastric adenocarcinoma, thyroid carcinoma, and pleomorphic glioblastoma, with metastasis and shorter time to recurrence [68]. In some solid tumors, including lung cancer, pheochromocytoma, hepatocellular carcinoma, bladder urothelial carcinoma, cervical squamous cell carcinoma, and gastric adenocarcinoma, adenosine pathway components are particularly overexpressed, including A2A and A2B. It is expected that these cancers may respond well to drugs targeting the eADO pathway [69]. As showed in Fig. 2, the expression levels of CD39, CD73 and A2AR were higher in several tumor types than their adjacent normal tissues. Moreover, the activation of CD39/CD73/adenosine-A2AR pathway is closely associated with an immunosuppressive TME and poor prognosis of cancer patients [5]. Therefore, CD39 and CD73 are indispensable for the development, differentiation, migration, and invasion of cancer cells [70–72]. Importantly, high expression levels of CD39 and CD73 have been associated with immune evasion of cancer cells as they can promote the infiltration of MDSCs and Tregs in tumor tissue [73]. Moreover, the activation of adenosine/A2AR signalling promoted Treg cell proliferation and the secretions of immune-suppressive factors (including TGFβ and IL-10) and upregulated the expression of immune-checkpoint receptors (such as PD-1, CTLA4 and LAG3), which mediated immunosuppression TME in tumor tissue and immune escape of cancer cells [74–76].

Given the hypoxic and inflammatory nature of many solid tumors, multiple components of the adenosinergic pathway are upregulated in malignant tissues compared with the respective non-malignant tissues [77]. The CD39 /CD73/A2AR signaling pathway has been shown to be associated with poor cancer prognosis. In addition, CD39 and CD73 are also involved in the formation of new lymphatic vessels around tumors and progression of malignant tumors such as breast carcinoma, multiforme glioblastoma and chronic lymphocytic leukemia [78–80]. Importantly, the blockade of adenosine/A2AR pathway resulted in the enhancement of cancer chemotherapy and immunotherapy in numerous cancer types including lung adenocarcinoma, renal clear cell carcinoma, pheochromocytoma and paraganglioma [43, 81–83]. Elevated levels of CD39 have also been found in tumors resected for hepatocellular carcinoma, gastric carcinoma, and head and neck squamous cell carcinoma, where higher expression is associated with the likelihood of recurrence after surgery and/or poor overall survival. In addition, FoxP3+ Tregs expressing CD39 were found to be better than FoxP3+ Tregs alone in predicting gastric cancer survival and time to recurrence of HCC [84]. Interestingly, in rectal adenocarcinoma, a combination of CD39 and CD73 expression provided better prognostic value, with CD73^hi^CD39^lo^ and CD73^lo^CD39^hi^ tumors showing worse and best outcomes, respectively [85]. CD73 may also predict better response to PD-1/PD-L1 targeted therapy, as it is strongly associated with PD-L1 expression in gastrointestinal neuroendocrine tumors [86]. As noted by Antonioli et al., the integration of CD73 and CD39 in prognostic assessment may contribute to enhanced stratification that helps determine the ideal therapeutic strategy in terms of adenosine energy axis’s contribution to cancer progression [87]. And in a smaller cohort of head and neck squamous cell carcinoma, Vogt et al. showed that while hypomethylation of NT5E was associated with worse outcomes, hypomethylation of ADORA2A was associated with longer overall survival [88]. Similarly, et al. found the opposite prognostic value of tumor CD73 and A2A protein expression in two coves of patients with non-small cell lung cancer or lung adenocarcinoma, where CD73 and A2A predicted poorer and better outcomes, respectively, and further studies are needed to better understand the effect of adenosine receptor expression on cancer prognosis [89].

## CD39/CD73/A2AR as a novel therapeutic target for combination therapy

Cancer immunotherapy including the PD-1/PD-L1 and CTLA-4 blockade regimens has achieved remarkable anticancer efficacy and long-term survival. However, only a small subset of cancer patients could benefit from the treatment. The fact that a large proportion of cancer patients do not respond suggest the presence of additional immunosuppressive pathway driving the immune evasion by the non-responding tumors [90, 91]. So that the CD39/CD73/A2AR signaling pathway appears to be an attractive target. In solid tumors, abundant ATP is released from the dying cells due to necrosis. CD39 and CD73 are highly expressed in numerous cancer types and also by the infiltrating immune cells. A2AR is expressed in the infiltrating immune cells [92, 93]. Thus, an immunosuppressive environment is reshaped in the TME by the accumulated adenosine to blunt the cancer immune surveillance. In fact, therapeutic targeting of the adenosine signaling has been proposed to enhance the efficacy of other existing cancer immunotherapy [94].

### Targeting the CD39/CD73/A2AR pathway

Small-molecule inhibitors and monoclonal antibodies targeting CD39, CD73 and A2AR have been developed for cancer therapy [95]. Generally speaking, monoclonal antibodies (mAb) are macromolecules and they may not penetrate well into solid tumors. In contrast, small molecules could cross physiologic barriers, such as plasma membrane and the blood–brain barrier, more easily. Thus, small-molecule inhibitors could achieve better exposure in the TME [96].

In various tumor models, a CD39-targeting mAb has been shown to inhibit the CD39 enzymatic activity on tumor surface and effectively suppress metastasis [97]. In a lung cancer model, another anti-CD39 mAb was shown to upregulate the expression of CD107a in infiltrating NK cells and promote IFN-γ release to kill cancer cells [98]. ES014 is an anti-CD39/TGF-β bispecific mAb. It was reported to simultaneously inhibit the enzymatic activity of CD39 and neutralize autocrine/paracrine TGF-β, which represent the two major immunosuppressive mechanisms in the TME. Therefore, ES014 could restore anti-tumor immunity by increasing the extracellular levels of the pro-inflammatory ATP, and inhibiting the accumulation of the immunosuppressive adenosine and TGF-β within the TME. Blockade of CD73 by the antagonistic CD73 mAb (3F7) has been shown to significantly delay tumor growth and inhibit metastasis in a 4 T1 breast tumor–bearing mouse model [99]. Moreover, it has been reported that anti-CD73 antibodies could enhance the anticancer effect of both anti-CTLA-4 and anti-PD-1 immunotherapy in multiple tumor-bearing mouse models. These studies also demonstrated that CD73 can inhibit antitumor leukocytes and interfere with adenosine generation to suppress tumor metastasis [100]. In a clinical trial, an anti-CD73 mAb (MEDI9447) with or without durvalumab (PD-L1 mAb) was reported to downregulate CD73 expression on peripheral T cells in 66 pancreatic and colorectal cancer patients, which was associated with an increase in cytotoxic T-cell infiltration [101]. Recently, Pe et al. found that IPH5201 (anti-CD39 mAb) and IPH5301 (anti-CD73 mAb) could efficiently block the hydrolysis of immunogenic ATP into immunosuppressive adenosine by specifically targeting human membrane-associated and soluble forms of CD39 and CD73, respectively. Importantly, IPH5201 and IPH5301 were shown to promote antitumor immunity by stimulating DCs and macrophages and by restoring the activation of T cells isolated from cancer patients [102].

On the other hand, a few small molecule CD39 or CD73 inhibitors are also underway in clinical trials. ES002023 is a CD39 inhibitor which restores antitumor immunity by stabilizing the pro-inflammatory extracellular ATP (eATP) and interfering with synthesis of the immunosuppressive adenosine within the TME (NCT05075564). AB680 is a highly potent, reversible and CD73-selective inhibitor. In preclinical studies, AB680 exhibited favorable pharmacokinetic properties. It is currently being evaluated in phase I clinical trials [103]. PSB-1248937 is another highly potent CD73 inhibitor recently developed but it is not absorbed well by the oral route [104]. There has been extensive search for small molecule CD39/73 inhibitors from natural compounds. Ellagic acid was recently identified as a lead compound for CD39 and CD73 dual inhibitor because of its low cytotoxicity to normal cells [105]. A few allosteric CD73 inhibitors that target the dimer interface have been identified by virtual screening [106]. By exploiting the binding mode of the human protein CD73 with α,β-methylene-ADP, Du et al. designed a series of novel effective small-molecule CD73 inhibitors. Among these CD73 inhibiting drug candidates, OP-5244 was shown to be highly potent and it can be taken orally with high bioavailability [18].

The accumulating preclinical researches demonstrated that the inhibition of A2AR activation can significantly increase antitumor immunity [107]. A2AR inhibitors have been shown to increase antitumor effects by boosting the effector function of cytotoxic lymphocytes and blocking the recruitment and polarization of immunosuppressive immune cells in the TME [108]. A novel A2AR antagonist CPI-444 has been shown to reduce the expression of multiple checkpoint pathways (including PD-1 and LARG-3) on CD8+ effector T cells and CD4+ regulatory T cells. Importantly, A2AR inhibition was found to exhibit the most pronounced effects during CD8+ effector T cell activation, thus remarkably reducing PD-1 and LAG-3 expression at the draining lymph nodes of tumor bearing mice [109]. Mechanistically, it has been demonstrated that the enhancement of IFN-γ production by the adoptively transplanted T lymphocytes contributes to the therapeutic benefit of A2AR antagonism. It is also noteworthy that A2AR antagonism could enhance antitumor immunity regardless of the tumor’s anatomical location and it could provide long-lasting tumor-specific memory [110].

As the adenosine-A2AR pathway is triggered by the binding of adenosine to A2AR to subsequently inhibit T-cell proliferation and function, a few small molecule inhibitors were designed to specifically interfere with the interaction between adenosine and A2AR. The blockage of the binding by ciforadenant and the A2AR inhibitor were reported to restore T-cell signaling, IL-2 and IFN-γ production [57, 111, 112]. AZD4635, a high-affinity oral A2AR antagonist, could reverse T-cell inhibition induced by the treatment with the adenosine analog 5′-*n*-ethylcarboxylated adenosine in vitro and in vivo [113]. It is currently on phase I clinical trials in patients with a variety of solid tumors [114]. The A2AR antagonist SCH58261 and PBF-509 were shown to block the MSC-mediated suppression of T-cell proliferation almost completely, thereby reactivating the antitumor immune response [115, 116]. We summarizes the various mAbs and small molecule targeting agents of CD39/CD73/A2AR that are currently in clinical trials for cancer therapy in Table 1.

**Table 1:** Investigation of monoclonal antibodies or small molecule inhibitors targeting the CD39/CD73/A2AR pathway in clinical trials. (https://clinicaltrials.gov/)

Agent/drug	Company	Mechanism	Phase	NCT number
JS019	Suzhou Kebo Ruijun Biotechnology Co, Ltd	Anti-CD39 monoclonal antibody	Phase I	NCT05508373
ES014	Elpiscience Biopharma, Ltd.	Anti-CD39/TGF-β bispecific antibody	Phase I	NCT05381935
PUR001	Purinomia Biotech, Inc.	Anti-CD39 monoclonal antibody	Phase I	NCT05234853
IPH5201	MedImmune LLC	CD39 antagonist	Phase I	NCT04261075
SRF617	Surface Oncology	CD39 antagonist	Phase I	NCT04336098
ES002023	Elpiscience Biopharma, Ltd.	CD39 antagonist	Phase I	NCT05075564
TTX-030	Trishula Therapeutics, Inc.	CD39 antagonist	Phase I	NCT03884556
PT199	Phanes Therapeutics	Anti-CD73 monoclonal antibody	Phase I	NCT05431270
IPH5301	Institut Paoli-Calmettes	Anti-CD73 antibody	Phase I	NCT05143970
TJ004309	I-Mab Biopharma US Limited	Anti-CD73 antibody	Phase II	NCT05001347
JAB-BX102	Jacobio Pharmaceuticals Co., Ltd.	Anti-CD73 monoclonal antibody	Phase I	NCT05174585
CPI-006	Corvus Pharmaceuticals, Inc.	Anti-CD73 antibody	Phase I	NCT03454451
AK119	Akeso	Anti-CD73 antibody	Phase I	NCT05173792
Sym024	Symphogen A/S	Anti-CD73 antibody	Phase I	NCT04672434
IBI325	Innovent Biologics (Suzhou) Co. Ltd.	Anti-CD73 antibody	Phase I	NCT05119998
Dalutrafusp (GS-1423)	Gilead Sciences	Anti-CD73-TGFβ-Trap bifunctional Antibody	Terminated	NCT03954704
HLX23	Shanghai Henlius Biotech	CD73 antagonist	Phase I	NCT04797468
AB680	Arcus Biosciences, Inc	CD73 antagonist	Phase I	NCT04104672
LY3475070	Eli Lilly and Company	CD73 antagonist	Phase I	NCT04148937
MEDI9447 (oleclumab)	AstraZeneca	CD73 antagonist	Phase I	NCT03736473
NZV930	Novartis Pharmaceuticals	CD73 antagonist	Phase I	NCT03549000
INCA 0186	Incyte Corporation	CD73 antagonist	Phase I	NCT04989387
BMS-986179	Bristol-Myers Squibb	CD73 antagonist	Phase I	NCT02754141
ORIC-533	ORIC Pharmaceuticals	CD73 antagonist	Phase I	NCT05227144
TT-10	Tarus Therapeutics, Inc.	A2AR antagonist	Phase II	NCT04969315
Ciforadenant (CPI-444)	M.D. Anderson Cancer Center	A2AR antagonist	Phase Ib/II	NCT05501054
PBF-509	Palobiofarma SL	A2AR antagonist	Phase I	NCT02403193
Taminadenant (NIR178)	Novartis Pharmaceuticals	A2AR antagonist	Phase II	NCT03207867
Inupadenant (EOS100850)	iTeos Therapeutics	A2AR antagonist	Phase I	NCT05117177
PBF-999	Palobiofarma SL	A2AR antagonist	Phase I	NCT03786484
CS3005	CStone Pharmaceuticals	A2AR antagonist	Phase I	NCT04233060
INCB106385	Incyte Corporation	A2AR antagonist	Phase I	NCT04580485
EXS21546	Exscientia Limited	A2AR antagonist	Phase I	NCT04727138
Etrumadenant (AB928)	Arcus Biosciences, Inc	A2AR and A2BR antagonist	Phase II	NCT04262856
AZD4635	AstraZeneca	A2AR antagonist	Phase I	NCT04478513

### Combination of CD39/CD73/A2AR inhibitors with other therapies

The combination of CD39/CD73/A2AR mAbs or small molecule inhibitors with conventional chemotherapy or other immunotherapies have been investigated in clinical trials on patients with advanced cancer [117, 118]. Additive and even synergistic anticancer effects were achieved in the combination of two distinct antitumor mechanisms. We summarizes the clinical investigations on combination of CD39/CD73/A2AR targeting mAbs or small molecule inhibitors with other cancer treatment modalities in Table 2. Remarkable inhibition of tumor initiation, growth, and metastasis were observed.

**Table 2:** Combinations of CD39/CD73/A2AR inhibitors and other cancer therapies under investigation in clinical trials (https://clinicaltrials.gov/)

Combination	Company	Mechanism	phase	NCT number
Combination SRF617 with pembrolizumab gemcitabine albuminbound paclitaxel	Surface Oncology Merck Sharp & Dohme LLC	CD39 antagonist with chemotherapy	Phase I	NCT04336098
Combination TTX-030 with immunotherapy and/or chemotherapy	Trishula Therapeutics, Inc. AbbVie	Anti-CD39 antibody with immunotherapy	Phase I	NCT04306900
Combination SRF617 with AB928 (Etrumadenent) and AB122 (zimberelimab)	Surface Oncology Arcus Biosciences, Inc	Anti-CD39 antibody with A2AR and A2BR antagonist	Phase I	NCT05177770
Combination IPH5301 with chemotherapy and trastuzumab	Institut Paoli-Calmettes Innate Pharma	Anti-CD73 antibody with chemotherapy	Phase I	NCT05143970
Combination AK119 with AK104	Akeso	Anti-CD73 antibody with chemotherapy	Phase I	NCT04572152
Combination IBI325 with sintilimab	Innovent Biologics (Suzhou) Co. Ltd.	Anti-CD73 antibody with chemotherapy	Phase I	NCT05119998
Combination oleclumab with gemcitabine, nab-paclitaxel, durvalumab	M.D. Anderson Cancer Center	Anti-CD73 antibody with chemotherapy	PhaseII	NCT04940286
Combination dalutrafusp (GS-1423) with mFOLFOX6 regimen	Gilead Sciences	Anti-CD73-TGFβ-Trap antibody with chemotherapy	Phase I	NCT03954704
Combination LY3475070 with pembrolizumab	Eli Lilly and Company Merck Sharp & Dohme LLC	CD73 antagonist with immunotherapy	Phase I	NCT04148937
Combination BMS-986179 with nivolumab (BMS-936558)	Bristol-Myers Squibb	CD73 antagonist with immunotherapy	Phase II	NCT02754141
Combination INCA00186 with INCB106385 and/or retifanlimab	Incyte Corporation	CD73 antagonist with immunotherapy	Phase I	NCT04989387
Combination TJ004309 with atezolizumab	I-Mab Biopharma US Limited I-Mab Biopharma Co. Ltd.	Anti-CD73 antibody with immunotherapy	Phase II	NCT05001347
Combination JAB-BX102 with pembrolizumab	Jacobio Pharmaceuticals Co., Ltd.	Anti-CD73 antibody with immunotherapy	Phase II	NCT05174585
Combination PT199 with an anti-PD-1 monoclonal antibody	Phanes Therapeutics	Anti-CD73 antibody with immunotherapy	Phase I	NCT05431270
Combination NZV930 with PDR001	Novartis Pharmaceuticals Novartis	Anti-CD73 antibody with immunotherapy	Phase I	NCT03549000
Combination Sym024 with Sym021	Symphogen A/S	Anti-CD73 antibody with immunotherapy	Phase I	NCT04672434
Combination oleclumab (MEDI9447) with AZD4635	MedImmune LLC	Anti-CD73 antibody with A2AR antagonist	Phase Ib/II	NCT03381274
Combination CPI-006 with ciforadenant or pembrolizumab	Corvus Pharmaceuticals, Inc	Anti-CD73 antibody with A2AR antagonist	Phase I	NCT03454451
Combination inupadenant (EOS100850) with Chemotherapy	iTeos Belgium SA iTeos Therapeutics	A2AR antagonist with chemotherapy	Phase II	NCT05403385
Combination INCB106385 with immunotherapy	Incyte Corporation	A2AR antagonist with immunotherapy	Phase I	NCT04580485
Combination NZV930 with PDR001 and /or NIR178	Novartis Pharmaceuticals Novartis	A2AR antagonist with immunotherapy	Phase I	NCT03549000
Combination lpilimumab, nivolumab with ciforadenant (CPI-444)	M.D. Anderson Cancer Center	A2AR antagonist with immunotherapy	phase I/II	NCT05501054
Combination NIR178 with PDR001	Novartis Pharmaceuticals Novartis	A2AR antagonist with immunotherapy	Phase II	NCT03207867
**Combination taminadenant with PDR001**	Palobiofarma SL Novartis H. Lee Moffitt Cancer Center and Research Institute	A2AR antagonist with immunotherapy	Phase I	NCT02403193
Combination DFF332, spartalizumab with taminadenant	Novartis Pharmaceuticals Novartis	A2AR antagonist with immunotherapy	Phase I	NCT04895748
Combination AZD4635 with durvalumab or oleclumab (MEDI9447)	AstraZeneca	A2AR antagonist with anti-CD73 antibody	Phase II	NCT04089553

Combing CD39/CD73/A2AR with other therapies is an attractive therapeutic strategy for cancer treatment. Targeting CD39/CD73/A2AR with blocking antibodies or small-molecule inhibitors in combination with other therapies such as immune checkpoint blockade and chemotherapy is a rational strategy to enhance therapeutic benefit

#### Combination of inhibitors targeting two members of the CD39/CD73/A2AR pathway

The adenosine-A2AR pathway consists of different components to convert ATP into the immunosuppressive adenosine. The disruption of individual member of the pathway and their combinations could give rise to different biological effects [119]. Targeted inhibition of A2AR and CD73 was shown to produce synergistic inhibition on tumor growth. The combination of sodium polyoxotungstate (small molecule CD73 inhibitor) and AZD4635 (A2AR antagonist) was found to block the adenosine pathway, thereby activating immune cells, increasing INF-γ production, and reducing the abundance of Treg cells [114]. On the other hand, the combination of IPH5201 (anti-CD39 mAb) and IPH5301 (anti-CD73 mAb) was reported to inhibit the production of adenosine, and subsequently reducing T cell inhibition in a co-culture system of myeloma and stromal cells in vitro [102]. In combination Oleclumab (MEDI9447,anti-CD73 antibody) with AZD4635(A2AR inhibitor), numbers of participants show Dose-limiting Toxicities (DLTs) and numbers of participants show Treatment Emergent Adverse Events (TEAEs) and Treatment Emergent Serious Adverse Events (TESAEs)(NCT03381274).

#### Combinations of CD39/CD73/A2AR inhibitor with other immunotherapies

The combination of CPI-444 (small molecule A2AR antagonist) and atezolizumab (anti-PD-L1 mAb) was reported to induce more durable anticancer response and more cytotoxic T-cell infiltration in TME than atezolizumab alone [112, 120]. In a recent clinical study, there were substantially more NSCLC patients achieving stable disease when treated with the combination of NIRI178 (A2AR antagonist) and spartalizumab (anti-PD-1 mAb) (14 out of 25) than treatment with apartalizumab alone (7 out of 25) [121]. Importantly, NIR178 with and without spartalizumab was well tolerated in all patients with advanced NSCLC [121]. Similarly, in another clinical trials on patients with advanced metastatic castration-resistant prostate cancer, the combination of AZD4635 (A2AR antagonist) and durvalumab (anti-PD-L1 mAb) was shown to produce more tumor responses (6 out of 37 patients) than treatment with durvalumab alone (2 out of 39 patients) [122]. These clinical data suggests that the inhibitor of CD39/CD73/A2AR pathway can enhance the efficacy of immune checking point inhibitor (ICI) in advanced solid tumorsIn combination PT199 with an anti-PD-1 monoclonal antibody, no loss of inhibition or “hook effect” is observed at a higher concentrations. Hence, PT199 is expected to increase antitumor immune activation, especially in combination with PD-1 pathway inhibition, and thus offer a new treatment option for cancer patients (NCT05431270).

#### Combinations of CD39/CD73/A2AR inhibitors with other Cancer therapies

The combination of photodynamic therapy and conventional chemotherapy is a promising strategy for destroying cancers that are either under the skin or in the lining of organs reachable by a light source. However, photodynamic therapy is not effective to treating metastatic diseases when tumor cells have already spread [123]. Jin et al. proposed that the combination of anti-CD73 mAb with chemo-photodynamic therapy can synergistically enhance the antimetastatic effects by boosting T cell–mediated antitumor immunity [123]. This approach has been investigated in animal model of metastatic triple-negative breast cancer. While the combination of photodynamic therapy and chemotherapy gave rise to strong antitumor effect and produced immunogenic cell death, the addition of anti-CD73 mAb could assure sufficient immune checkpoint blockade in the tumors by blocking the adenosine pathway [123]. More importantly, this combination strategy was also shown to prevent abscopal tumor metastasis by inducing systemic cytotoxic T cell response via CD73 blockade [123]. However, in a clinical study investigating the combination of IPH5301 (anti-CD73 mAb) with chemotherapy or trastuzumab, dose limiting toxicity of IPH5301 was observed in the combination group. Moreover, similar antitumor response was achieved in the IPH5301-paclitaxel-trastuzumab combination group and the IPH5301 monotherapy group. And the clinical trail of combination dalutrafusp (GS-1423) with mFOLFOX6 regimen was terminated . The decision to discontinue the study was made based on the totality of the clinical, pharmacokinetic, and pharmacodynamic findings (NCT03954704).

It has been proposed that inhibition of adenosine-A2AR pathway could promote the abundance and infiltration of cytotoxic T cells into tumors [124]. Given that the cytokine IL-7 signaling could facilitate the accumulation of tumor-associated CD8+ T cells by hindering adenosine-mediated immunosuppression, the combination of IL-7 modulator and adenosine-A2AR inhibitors have been evaluated for treatment of solid tumors [125]. Newton et al. reported the specific knockdown of A2AR by a lipid nanoparticle-based system to promote the chemotaxis of head and neck cancer memory T cells into the solid tumor [126]. On the other hand, the combination of A2AR antagonists with NK-cell therapy has also been shown to enhance antitumor immunity. DC-based cancer vaccines represent another promising approach for cancer immunotherapy. While efficacy from DC vaccines relies heavily on antitumor T-cell responses [127], cancer cells could utilize the adenosine-A2AR pathway to escape from the antitumor immunity of DC vaccines. So Arabet et al. investigated the potential therapeutic application of combining DC vaccine with inhibitor of the CD39/CD73/A2AR pathway [128]. Apart from promoting angiogenesis and anti-inflammatory activities, CD39 also plays an important role in regulating thrombogenesis to provide adequate blood supply to tumor cells. It was known that tumor cells, endothelial cells, and tumor-infiltrating immune cells express CD39, which suppresses anti-tumor immune responses and promotes tumor growth [129]. Collectively, combination of inhibitor of CD39/CD73/A2AR pathway and cancer immunotherapy is emerged as a novel strategy for treating solid tumors.

## Biomarkers of the CD39/CD73/A2AR pathway in Cancer

Recent studies have shown that CD73 is overexpressed in solid tumors such as ovarian, gastric, breast, colorectal cancer [130]. In clinical studies, tumoral CD73 expression was negatively correlated with immune cells infiltration of tumors, worse disease-free survival rate, and poorer overall survival in cancer patients [131]. In prospective clinical trial investigating adenosine pathway inhibitors, inhibition of the CD39/CD73/A2AR pathway was shown to increase immune cell activation, expand T cell repertoire in peripheral blood, and also increase T cell infiltration in tumor biopsy samples [132]. There have been extensive studies investigating pharmacodynamics biomarkers that could predict the clinical responses of adenosine inhibitors. We summarizes the more promising biomarkers used to predict the efficacy of adenosine pathway inhibitors in various cancer types in Table 3.

**Table 3:** Biomarkers related to the CD39/CD73/A2AR pathway in cancer

Name	Biomarkers	Reference
Adenosine gene signature(8 genes)	CXCL1,2,3,5,6,8,PTGS2 and IL-1β recognized as adenosine signature,was positively correlated with adenosine levels.	[96], [133]
Adenosine Signaling Signature(14 genes)	In human cancer, the gene expression of PPARG, CYBB, COL3A1, FOXP3, LAG3, APP, CD81, GPI, PTGS2, CASP1, FOS, MAPK1, MAPK3, CREB1 correlated with A2AR signaling.	[134]
Inflammatory cytokines or molecules	The expression level of CD39 and CD73 are upregulated by various inflammatory cytokines or molecules, including type I interferons, IL-2, IL-1β, IL-6,IL-27, tumour necrosis factor (TNF), prostaglandin E2 or aryl hydrocarbon receptor agonists.	[51]
Tenascin C and EGFR	CD73 directly bind and activate transcin C and EGFR to promote tumor cell growth,adhesiveness and invasiveness.	[135]
Hypoxia-inducible factor (HIF1)	CD39, CD73 and adenosine receptor, including A2A and A2B, are regulated by HIF, which also can inhibit the activity of adenylate kinase and ENTs, inducing the accumulation of adenosine and immunosupressive response in TME.	[73]
p-CREB and p-S6	p-CREB and p-S6 may represent useful pharmacodynamic and efficacy biomarkers of immunotherapies targeting Adenosine.	[92]
TGFβ	In immnue cells, such as T cells, NK cells,myeloid cells,tumour cells,fibroblasts and endothelial cells, the expression level of CD39 and CD73 are upregulated by TGFβ.	[73]
ADA (Adenosine deaminas)	ADA serves as a diagnostic biomarker in lung malignancies. May be valuable to predict which patients may respond better to treatments of blocking adenosine production or signaling.	[136]
LYVE1,PDPN,VEGFC	LYVE1, PDPN and VEGFC positively correlated with the gene expression of ADOA2AR, NT5E and ENTPD1,respectively coding A2AR, CD73, CD39, thereby influencing the adenosine production in several human cancers.	[137]
Intercellulae adhesion molecule 1(ICM-1)	Adenosine suppressed the upregulation of ICAM-1 mediated by IL-18 on human monocytes and it eliminated the production of IL-12, IFN-γ and TNF-α mediated by the enhancement of IL-18.	[138]

Biomarkers that were identified adenosine in CD39/CD73/A2AR pathway remain to be defined. *EGFR* Epidermal Growth Factor Receptor, *p-CREB* Phospho- CAMP-response element-binding, *CXCR2* C-X-C motif chemokine receptor 2, *TGFβ* Transforming growth factor-β, *LYVE1* Lymphatic Vessel Endothelial receptor-1, *PDPN* Podoplanin, *VEGFC* Vascular endothelial growth factor C

Since adenosine is metabolized rapidly and its half-life in plasma is only about 10s, it is difficult to directly measure the level of adenosine in patient samples. Therefore, adenosine cannot be used as a biomarker by directly measuring its level in tumor specimens [70]. On the other hand, adenosine-related gene expression profiles were found to correlate well with adenosine levels in tumors. Thus, the profile of adenosine-related gene expression may be used as potential biomarkers to predict treatment response from adenosine-A2AR inhibitors. A recent study revealed that the expression of a group of genes related to myeloid cell biology and inflammation was positively correlated with adenosine levels [96]. A set of 8 genes (including CXCL1, CXCL2, CXCL3, CXCL5, CXCL6, CXCL8, PTGS2 and IL-1β) was subsequently coined as the “Adenosine Gene Signature” (AdenoSig) to identify patients likely to respond to treatment with the A2AR antagonist (Ciforadenant) [96, 133]. Meanwhile, Sidders et al. proposed another genomic signature termed the “Adenosine Signaling Score” consisting of 14 genes (PPARG, CYBB, COL3A1, FOXP3, LAG3, APP, CD81, GPI, PTGS2, CASP1, FOS, MAPK1, MAPK3, CREB1), which exhibited good correlation with A2AR signaling in human cancers and could be used to predict immunotherapeutic response [134]. The Adenosine Signaling Score is directly proportional to the concentration of adenosine and it was significantly reduced in A2AR-knockout models. Interestingly, while the AdenoSig and Adenosine Signaling Score only share a single gene in common, they are highly correlated to each other in several solid tumors [133].

On the other hand, decreased adenosine deaminase (ADA) levels in brochoalveolar lavage (BAL) has been used as a diagnostic biomarker for lung cancer. As it is often difficult to obtain sufficient lung tissue from cancer patients for proper diagnosis, ADA levels in BAL could be used as an auxiliary parameter for making malignancy and histopathological diagnoses in conjunction with radiological and clinical findings [139]. It is noteworthy that immunosuppressive functions of CD14^high^ CD163^high^ CD39^high^ macrophages, as well as the secretion of IL-10, were diminished by ADA, thus allowing the measurement of ADA to reflect the status of immunosuppression in the TME [136, 140].

In addition, it has been reported that CD73 expression is upregulated in response to specific oncogenic mutations, including TP53, EGFR and RAS [73]. The expression of CD73 was also correlated well with genes altered by hypoxic and tissue-repair responses, including TGFβ and epithelial-to-mesenchymal transition genes [73]. In various solid tumors, including breast, colorectal, ovarian and pancreatic cancers, cancer-associated fibroblasts (CAFs) constitute the prominent cell population with high expression of CD39 and CD73, which facilitate a feedforward circuit to enforce the CD73 immune checkpoint and maintain an immunosuppressive TME [141]. Furthermore, activation of the EMT was shown to increase CD73 expression and thus eADO receptor signalling, which further enhances the EMT phenotype [72]. Recently, Smyth et al. reported that adenosine signaling could impair the immune effect of peripheral T cells and tumor-infiltrating lymphocytes (TILs) via a A2AR/PKA/mTORC1 signalling pathway [92]. In this study, phosphoflow staining of CREB and S6 proteins was used to assess the influence of adenosine/adenosine receptor on the activation of the PKA and mTOR pathways, respectively Therefore, p-CREB and p-S6 may be used as useful pharmacodynamic and efficacy biomarkers to predict therapeutic response of adenosine-targeting immunotherapies [92]. In summary, various genetic signatures and signalling molecules could be used to select individual cancer patients who may benefit from adenosine-targeting therapy.

## Conclusions

The immunosuppressive TME is the major hindrance to successful cancer immunotherapy, which must be overcome in order to achieve robust and durable antitumor response. It has been shown that the purinergic signaling axis contributes to tumor-mediated immunosuppression. The CD39/CD73/adenosine/A2AR signaling is emerging as a promising therapeutic target because adenosine produced by the purine nucleoside in TME can strongly inhibit the immune system. The intratumoral production of adenosine is dependent on the sequential catabolism of ATP by two ectonucleotidases, CD39 (from ATP to AMP) and CD73 (from AMP to adenosine). It is increasingly evidence that CD39/CD73/A2AR pathways play a crucial role in regulating immune responses, both in normal physiology and in pathological states. Importantly, the inhibition of CD73 eliminates a major pathway for adenosine production within the TME and can reverse the immunosuppressive effect mediated by adenosine. Targeting CD39/CD73/A2AR with blocking antibodies or small-molecule inhibitors has exhibited strong antitumor efficacy. In addition, the simultaneous inhibition of CD73 and A2AR was shown to give rise to synergistic effect. Recent findings in the field advocates the development of specific inhibitors targeting CD39/CD73/A2AR to potentiate cancer immunotherapies.

Although both in vitro experiments and animal model studies have confirmed the great potential of targeting CD39/CD73/A2AR pathways for cancer treatment, translating these results into clinical practice will require a deeper understanding of how adenosine regulates the cancer microenvironment. However, one of the deficit in our knowledge is that adenosine promotes cancer growth through its effects on cancer stroma, the direct effects of adenosine on cancer cells are variable. It is also crucial to master a variety of detailed research methods in order to analyze tumor inhibition of adenosine pathways mediated by cancer stroma, such as conditional deletion of adenosine receptors or metabolic enzymes in immune cells or endothelial cells, silencing adenosine receptors or metabolic enzymes in xenograft or allograft prior to inoculation and using a three-dimensional cell culture model that contains cancer cells that constitute their microenvironment. Another factor, the potential use of adenosine drugs in cancer - the intrinsic impact of the adenosine system depends on several factors, including the type of cancer, adenosine receptor subtypes expressed by cancer cells and studies of proliferation, apoptosis or metastasis, such as the fact that a particular tumor may express multiple adenosine receptors, adenosine therapy should take into account these competing proliferative and antiproliferative (or pro-apoptotic and anti-apoptotic) roles of various receptors.

In addition to preclinical studies, clinical studies using adenosine drugs should also rely on a better understanding of specific tumors in humans. Biomarker-based tumor monitoring can guide such adenosine therapy, and these biomarkers may involve various adenosine receptors, metabolic enzymes, and uptake systems, for example, A2B receptor-dependent breast cancer with high expression of A2B receptor can be treated with A2B receptor antagonists. We anticipate that these approaches combined with the analysis of potential polymorphisms in the human adenosine system, will help us to realize the potential of adenosine therapy in the management of cancer patients. With lots of preclinical and clinical studies, the application of inhibitors of the CD39-CD73-A2AR pathway will be broadened and improved. Furthemore,the efficacy of the combination regimen with other immune checkpoint inhibitors has been established and evaluated in preclinical studies. In addition, recent preclinical studies have shown that the benefits of combining CAR T cell therapy with A2AR blocking are quite constructive, investigating such clinical trials and protocols are imminent. Since adenosine production depends on hypoxic conditions and cell renewal, blocking this pathway in combination with therapies that promote hypoxia and cell death within TME should be valuable. These include radiation therapy, which creates hypoxic conditions, and chemotherapy drugs, especially those that increase ATP release (known as “immunogenic chemotherapy”). The diversity of CD39/CD73/A2AR signaling pathway mediated immune mechanisms may indicate its wide application in clinical field.

## Data Availability

Not applicable.

## Abbreviations

CD39: Exonucleoside triphosphate diphosphate hydrolase 1
CD73: Ecto-5′-nucleotidase
A1R, A2AR, A2BR and A3R: Adenosine A1, A2A, A2B, A3 receptors
TME: Tumor microenvironment
DAMP: Danger-Associated Molecular Pattern
NK: Natural killer
DCs: Dendritic cells
MDSC: Myeloid-derived suppressor cells
Treg: Regulatory T cells
Th17: T helper 17 cells
Th1, Th2: T helper 1, 2 cell
Th Sp1: Transcription factor Sp1
Stat3: Signal transducer and activator of transcription 3
GFI1: Growth factor independence-1
IL-17: Interleukin 17
EGFR: Epidermal Growth Factor Receptor
p-CREB: Phospho- CAMP-response element-binding
CXCR2: C-X-C motif chemokine receptor 2
TGFβ: Transforming growth factor-β
LYVE1: Lymphatic Vessel Endothelial receptor-1
PDPN: Podoplanin
VEGFC: Vascular endothelial growth factor C
eATP: Extracellular ATP
HIF-1α: Hypoxia inducible factor 1 alpha subunit
CTLA4: Cytotoxic T-lymphocyte associated protein 4
LAG3: Lymphocyte activation gene 3 protein
IFN-γ: Interferon γ
PTGS2: Prostaglandin-endoperoxide synthase 2
PPARG: Peroxisome proliferative activated receptor, gamma
CYBB: Cytochrome b-245 beta chain
COL3A1: Collagen alpha-1(III) chain
FOXP3: forkhead box P3
APP: Amyloid Precursor Protein
GPI: Glucose-6-phosphate isomeras
CASP1: Caspase 1, apoptosis-related cysteine peptidase
MAPK: Mitogen-activated protein kinase
ADA: Adenosine deaminase
IL-10: Interleukin 10
CAFs: Cancer-associated fibroblasts
EMT: Epithelial-MesenchymalTransition
PD-1: Programmed cell death protein 1
PD-L1: Programmed cell death-Ligand 1
B7-H3: CD276, Recombinant Cluster Of Differentiation 276
TIM3: T cell immunoglobulin domain and mucin domain-3
TIL: Tumor Infiltrating Lymphocyte
eADO: Extracellular adenosine.
